# Supplementary material for: Synergistic effects of physical-mental mixed fatigue on badminton forehand smash performance
Source: PLoS One. 2026 Feb 25;21(2):e0337467. doi: 10.1371/journal.pone.0337467 (PMC12935304; doi:10.1371/journal.pone.0337467)
Supplement: Attachment — Submitted filename: Response to Reviewers.docx [file pone.0337467.s004.docx]

Response to Reviewers

Manuscript Title: Synergistic Effects of Physical-Mental Mixed Fatigue on Badminton Forehand Smash Performance

Manuscript Number: PONE-D-25-24801

Journal: PLOS ONE

Dear Editor and Reviewers,

We sincerely thank you for your valuable comments and constructive suggestions on our manuscript entitled "Synergistic Effects of Physical-Mental Mixed Fatigue on Badminton Forehand Smash Performance." We have carefully revised the manuscript according to each comment and suggestion provided by the editor and reviewers. All changes in the manuscript are highlighted in red for your easy reference.

Below, we provide point-by-point responses to each comment, along with explanations of how the manuscript has been revised accordingly. We hope that the revised version meets your expectations and look forward to your favorable consideration.

Editor Comment

We sincerely thank the editor for highlighting this point. We acknowledge that evaluating physical and mental fatigue separately would provide deeper mechanistic insight. However, the primary aim of this study was to simulate the real competitive scenarios where both types of fatigue often occur simultaneously. We now explicitly clarify this rationale in the Introduction and Discussion, and have emphasized in the limitations section that future studies should investigate physical and cognitive fatigue independently.

**Reviewer #1 – 1. Abstract: Add a sentence on practical implications (e.g., "Findings suggest training should integrate fatigue-mitigation strategies").**

**Response:** We appreciate this suggestion. We have now added a sentence to the end of the abstract to reflect the practical relevance of the findings: 'These findings suggest that coaches should integrate fatigue simulation and mitigation strategies into technical training to sustain performance under competitive conditions.'

**Reviewer #1 – 2. Introduction :The "dual-channel model" is introduced but not grounded in existing theories (e.g., Central Governor Model).**

**Response:** We appreciate this suggestion. We revised the Introduction to link our neuromuscular-cognitive dual-channel fatigue model with existing theoretical frameworks, including the Central Governor Model and psychobiological models of fatigue.

**Reviewer #1 – 2. Introduction : Justification for SAT : The speed-accuracy trade-off (SAT) is assumed; cite prior badminton-specific SAT evidence (e.g., Afzal et al., 2020). The necessity must be more**

**Response:** We appreciate this suggestion. We cited prior evidence supporting SAT in badminton, especially Afzal et al. (2020), in the Introduction and Discussion, strengthening the theoretical rationale.

**Reviewer #1 - 3. Methods**

**- Gender bias : Only 4 females (16.7%); no analysis of sex differences. - Justify gender imbalance or analyze sexes separately.**

**- Task inconsistency : Males used jump smashes, females stationary strokes—confounds biomechanical comparisons.**

**1. Methodology : Address gender bias and task standardization.**

**2. Discussion : Ground mechanistic claims in d	ata; avoid overinterpretation.**

**3. Writing : Reduce redundancy; clarify jargon.**

**Response:** Thank you for your suggestions above, and I will explain them to you one by one.

**1.Gender imbalance and task inconsistency**

Thank you for highlighting this important point. Upon review, we acknowledge that there was an error in the initial manuscript. In fact, both male and female participants performed the forehand jump smash under all testing conditions. We have corrected this description in the Methods section to accurately reflect the procedures. Additionally, the related statements in the Discussion have been revised to eliminate the incorrect explanation regarding task differences between genders.

**2.Mechanistic overinterpretation**

**Response:** Thank you for your suggestion. We reviewed and revised mechanistic statements to ensure they are supported by data or literature. Unsupported speculative phrases were removed or softened.

**3.Language and redundancy**

**Response:** Thank you for your suggestion. We revised the Introduction to remove repetitive statements and clarified technical terminology across the manuscript to improve clarity and conciseness.

**How can you predict how much was the effect of mental and physical fatigue. evaluating just combination of the seems insufficient for innovation as well as practical recommendation.**

**Response:** We clarified in the Introduction and Discussion that this study intentionally focused on mixed fatigue to reflect real-match scenarios. The Limitations section now suggests that future studies disentangle these components through factorial designs.

**Reviewer #2 - 1. In the introduction section, The author repeatedly emphasizes that "previous studies have examined physical fatigue and cognitive fatigue separately," a point reiterated across multiple paragraphs without advancing the argument, resulting in redundancy.**

**Response:** We appreciate this suggestion. We consolidated repeated statements about separately studied fatigue modalities into a single paragraph to improve focus and reduce redundancy.

**Reviewer #2 - . The current draft provides a reasonably broad overview of physical and mental fatigue, but its theoretical depth remains insufficient. The existing content primarily focuses on fatigue's impact on performance metrics (such as shot speed and accuracy) while neglecting discussions on fatigue-induced alterations in motor control and potential injury risks. Particularly in complex movements like the badminton jump smash—which combines high-speed leaps with precision striking—fatigue not only diminishes technical output but may also modify lower-limb kinetic patterns, exacerbating joint loading. This critical aspect has yet to receive due attention in the current introduction.**

**We recommend that the authors cite the study "Accurately and effectively predict the ACL force: Utilizing biomechanical landing pattern before and after-fatigue" (https://doi.org/10.1016/j.cmpb.2023.107761). This research demonstrates that fatigue significantly alters athletes' landing patterns, leading to markedly increased anterior cruciate ligament (ACL) forces, highlighting potential biomechanical injury risks under fatigued conditions. Additionally, "Analysis of Quadriceps Fatigue Effects on Lower Extremity Injury Risks During Landing Phases in Badminton Scissor Jump" (https://doi.org/10.3390/s25082536) further indicates that quadriceps fatigue in badminton induces reduced knee dynamic stability, increased valgus collapse, and other changes that elevate lower-limb injury risks. Incorporating these findings into the introduction would deepen the understanding of fatigue's mechanistic effects and strengthen the theoretical relevance and applied value of this study in the context of jump smash execution. By broadening the perspective—from isolated technical performance decline to biomechanical control and injury prevention—the manuscript would gain greater academic persuasiveness in the field of sports biomechanics.**

**Response:** We added a paragraph in the Introduction discussing how fatigue may alter neuromuscular control and increase injury risk, citing two recent studies on ACL loading and quadriceps fatigue in badminton.

**Reviewer #2 - 3. Insufficient physiological quantification of fatigue induction: The use of RPE and VAFS alone to assess fatigue levels is a limitation. These scales, while widely used, are susceptible to individual interpretation.**

**Response:** We appreciate this suggestion. We acknowledged this limitation in the Discussion and suggested future inclusion of objective indicators such as EMG, lactate, and HRV.

**Reviewer #2 - Male players executed jump smashes while female players performed stationary strokes. This introduces a clear biomechanical and metabolic disparity in task demands, yet no stratified analyses or justifications are provided.**

**Response:** We appreciate this suggestion. This overlaps with Reviewer #1's concern and was addressed similarly: explanation added in Methods and limitation noted in Discussion.

**Reviewer #2 - Effect size omission despite high significance: While the p-values suggest strong statistical significance, readers are left without a sense of how meaningful the observed changes are. Reporting effect sizes (e.g., Cohen’s d or partial eta squared) would significantly improve the interpretability of the findings.**

**Response:** We appreciate this suggestion. We calculated and reported effect sizes (partial eta squared and Cohen's d) in the Results section.

**Reviewer #2 - Superficial limitations discussion: The authors mention sample size and ecological validity but do not offer concrete suggestions for addressing these in future work. Including proposals such as wearable EMG, eye-tracking for decision-making, or real-match testing protocols would enrich the limitations section and demonstrate critical reflection.**

**Response:** We appreciate this suggestion. We expanded the Limitations section to include actionable suggestions: wearable EMG, eye-tracking systems, and match-simulated protocols to enhance ecological validity.

**We sincerely thank the editor and reviewers once again for their valuable time and insightful comments. Their suggestions have greatly improved the rigor and clarity of our manuscript. We hope the revised version meets the high standards of the journal and look forward to your favorable consideration!**
