# Supplementary material for: Synergistic effects of physical-mental mixed fatigue on badminton forehand smash performance
Source: PLoS One. 2026 Feb 25;21(2):e0337467. doi: 10.1371/journal.pone.0337467 (PMC12935304; doi:10.1371/journal.pone.0337467)
Supplement: Attachment — Submitted filename: Response_to_Reviewers_auresp_2.docx [file pone.0337467.s005.docx]

**Response to Reviewers**

Manuscript ID: PONE-D-25-24801R1

Title: Synergistic Effects of Physical-Mental Mixed Fatigue on Badminton Forehand Smash Performance

Dear Editor and Reviewers,

We sincerely thank you for the valuable time and effort you dedicated to reviewing our manuscript. We carefully considered each comment and have revised the manuscript accordingly. Below, we provide a detailed, point-by-point response. All changes have been incorporated into the revised version, with tracked changes for clarity.

Major Revisions

Reviewer #1, Comment 1: Provide more details on the randomization process for target-hitting tasks (e.g., frequency of signal changes, spatial distribution).

Response: We sincerely thank the reviewer for this important suggestion. In the revised manuscript, we have added a detailed description of the randomization process in the Physical and Mental Mixed Fatigue section. Specifically, light signals changed randomly every 2–4 seconds, with equal probability across four predefined target zones, ensuring unpredictability in both temporal and spatial dimensions.

Reviewer #1, Comment 2: Clarify how "successful shots" were defined during mental fatigue induction (e.g., accuracy thresholds, time constraints).

Response: We appreciate the reviewer’s constructive comment. We have now clarified in the Mental Fatigue Induction subsection that a shot was considered successful if it landed within ±5 cm of the designated target within 2 seconds after the visual signal appeared.

Reviewer #1, Comment 3: Please clarify how the effect of menstrual cycle has been considered for its most probable effect on variables.

Response: Thank you for highlighting this key methodological issue. In the revised Participants section, we have specified that all female participants reported their menstrual cycle phase, and testing sessions were scheduled during the follicular phase to minimize hormonal influence on performance outcomes.

Reviewer #1, Comment 4: Statistical analysis: How normality of distribution and other pretest requirements were checked before using parametric statistical tests?

Response: We greatly appreciate this suggestion. We have added a description of the preliminary statistical checks in the Statistical Analysis section. Specifically, Shapiro–Wilk tests were used to examine normality, while Levene’s test verified homogeneity of variances. All assumptions for parametric analysis were met.

Reviewer #1, Comment 5: Explanation for similarity of test with real badminton game is missing.

Response: We thank the reviewer for this valuable reminder. In the revised manuscript, we have added a statement clarifying the ecological relevance of the protocol, which incorporated rapid footwork, reactive shot-making, and unpredictable stimulus presentation, closely simulating the combined physical and cognitive demands of real badminton competition.

Reviewer #1, Comment 6: Expand the discussion on SAT mechanisms in badminton; reference dual-task interference theories.

Response: We sincerely appreciate this insightful suggestion. The Discussion has been expanded to incorporate dual-task interference theories, particularly Kahneman’s Capacity Model, which explains how limited attentional resources under moderate fatigue exacerbate speed–accuracy trade-offs (SAT) in badminton performance.

Reviewer #1, Comment 7: Discuss central and peripheral neurophysiological mechanisms, citing recent literature.

Response: We thank the reviewer for this excellent suggestion. The Discussion section now integrates both central (e.g., prefrontal cortex suppression) and peripheral (e.g., EMG alterations) mechanisms, supported by recent references, to provide a more comprehensive explanation of the observed effects.

Reviewer #1, Comment 8: Discuss the ecological validity of the lab-based protocol.

Response: We are grateful for this comment. The Limitations subsection has been updated to explicitly acknowledge the lack of competitive elements (e.g., audience presence, match pressure) in our laboratory protocol, which may influence ecological validity relative to real-game situations.

Minor Revisions

Reviewer #1, Minor Comment 1: Define acronyms (e.g., VAFS, RPE) at first use.

Response: Thank you for pointing this out. All acronyms are now defined at their first occurrence in the text.

Reviewer #1, Minor Comment 2: Standardize terminology (e.g., "smash speed" vs. "shuttlecock speed").

Response: We appreciate this careful observation. Terminology has been standardized, and “smash speed” is used consistently throughout the manuscript.

Reviewer #1, Minor Comment 3: Label axes clearly in Figures 3/4.

Response: Thank you for this suggestion. Axes in Figures 3 and 4 have been revised to clearly include appropriate units (RPE and VAFS as scores; smash speed in km/h; accuracy in points).

Reviewer #1, Minor Comment 4: Table 4 should include confidence intervals for mean differences.

Response: We agree with this important recommendation. Table 4 has been updated to include 95% confidence intervals for mean differences.

Reviewer #1, Minor Comment 5: Include explanations for abbreviations below tables.

Response: We thank the reviewer for this helpful reminder. Explanations of abbreviations (e.g., RPE) have now been added below all relevant tables for clarity.

Reviewer #2

We sincerely thank Reviewer #2 for confirming that all previous comments have been addressed and for supporting the revised version of our manuscript.

Closing Acknowledgment

We are deeply grateful to both reviewers and the editor for their constructive and insightful feedback. The revisions made in response to these comments have substantially strengthened our manuscript. We believe that the revised version now meets the standards of PLOS ONE, and we respectfully submit it for your further consideration.

Sincerely,

Meng Liu and co-authors
