# Supplementary material for: Synergistic effects of physical-mental mixed fatigue on badminton forehand smash performance
Source: PLoS One. 2026 Feb 25;21(2):e0337467. doi: 10.1371/journal.pone.0337467 (PMC12935304; doi:10.1371/journal.pone.0337467)
Supplement: Attachment — Submitted filename: Response_to_Reviewers_auresp_3.docx [file pone.0337467.s006.docx]

***Response to Reviewers***

**Dear Academic Editor and Reviewers,**

We sincerely thank you for your thoughtful and constructive comments, which have greatly helped us improve the quality and clarity of our manuscript. We have carefully revised the paper according to your suggestions. Below, we provide a detailed point-by-point response. All changes have been highlighted in the revised manuscript.

***Reviewer Comment 1 (Critical): Methodological Confound***

***Comment:*** The authors must add a justification in the Methods section for the different task protocols (jump smash vs. stationary stroke). A sentence such as: “Due to well-documented differences in average jump height and power output between elite male and female athletes in our recruitment pool, the female participants performed a restricted-step stationary stroke to ensure technical safety and movement standardization. While this limits direct biomechanical comparison, it allows for the assessment of within-subject fatigue effects on each athlete's maximal performance capability.” Additionally, they should acknowledge this as a study limitation in the Discussion.

***Response:*** We appreciate this insightful comment. We have added the suggested justification to the Methods section, clarifying the rationale behind the use of different task protocols for male and female participants. The new text reads as follows:

The test protocol required participants to perform 10 full forehand spikes, aiming at two geometric zones (212 × 40 cm zone control area) on the sideline of singles. To standardize biomechanical comparability, male participants executed the forehand jump smash, while female participants performed a restricted-step stationary stroke. Due to well-documented differences in average jump height and power output between elite male and female athletes in our recruitment pool, the female participants performed the stationary stroke to ensure technical safety and movement standardization. While this limits direct biomechanical comparison, it allows for the assessment of within-subject fatigue effects on each athlete’s maximal performance capability. The ball machine (EDIBO 3.0, launch frequency 3 Hz ± 0.5°, shuttlecock speed 70 ± 2 m/s, coordinate positioning error < 1 cm) was positioned 75–80 cm behind the doubles service line, projecting shuttles toward the court center with ascending trajectories at 3-second intervals using new shuttlecocks for each trial (Fig. 1). The performance assessment was explained to the participants before starting the test. During the test, participants were strongly encouraged and informed of their outcomes in order to maintain vigilance and concentration throughout the procedure.

***In addition, we have revised the Discussion (Limitations) section to acknowledge this methodological limitation:***

While laboratory settings control confounding variables, they fail to fully replicate competitive stressors (e.g., audience effects, real-time pressure), potentially underestimating mental fatigue impacts. Additionally, the absence of neurophysiological metrics (e.g., EEG/EMG) limits analysis of central-peripheral fatigue interactions, and the small sample size (n=24) of national-level athletes restricts generalizability to amateur populations. Moreover, due to well-documented differences in average jump height and power output between elite male and female athletes in our recruitment pool, female participants performed a restricted-step stationary stroke instead of a jump smash to ensure technical safety and movement standardization. While this protocol limits direct biomechanical comparison between genders, it allows reliable assessment of within-subject fatigue effects. Nevertheless, future studies may further explore gender-specific fatigue responses using larger, more balanced samples. Future studies should integrate field experiments with psychophysiological monitoring (e.g., wireless surface EMG, eye-tracking) to dynamically assess muscle activation patterns and visual search strategies under real-world fatigue conditions, alongside developing personalized interventions such as cognitive-physical cross-training.

***Reviewer Comment 2: Clarify Statistical Analysis***

***Comment:*** In the Data Processing section (Lines 139–140), it states ‘calculate the average speed by averaging 10 speed values.’ It should be clarified that this was done for each fatigue condition (Baseline, Mild, Moderate, Severe), resulting in four average speed values per participant.

***Response:*** We agree and have revised the sentence in the Data Processing section for clarity. The updated text now reads:

Every forehand smash, the speed at which the players hit the badminton is measured by radar (Stalker ATS, USA). The radar is located 3 meters behind the players with a height of 2.50 meters. In order to ensure that the speed data is recorded, the experimenter manually points the radar at the area aimed at by the player. All the data are recorded on a personal laptop. The shuttlecock speed was measured for each of the 20 smashes under every fatigue condition (Baseline, Mild, Moderate, and Severe). For each condition, the average stroke speed was calculated by averaging 10 consecutive valid speed values, resulting in four mean speed values per participant (see Figure 1 for radar positioning).

**This change ensures the procedure and the statistical logic are explicit and replicable.**

***Reviewer Comment 3: Minor Language and Consistency Edits***

***Comment:*** Line 124: ‘fatigue agreement’ should likely be ‘fatigue protocol.’ Ensure consistency in terminology, e.g., ‘mental fatigue’ vs. ‘cognitive fatigue’ are used interchangeably; choosing one would enhance clarity.

***Response:*** We have corrected “fatigue agreement” to “fatigue protocol” and standardized the terminology throughout the manuscript by consistently using “mental fatigue” instead of “cognitive fatigue.” These changes improve the precision and consistency of the text.

**We believe these revisions have substantially improved the manuscript’s clarity, methodological transparency, and consistency. We appreciate the reviewers’ valuable feedback and hope that our revisions meet their expectations.**

**Thank you very much for your time and consideration.**

Sincerely,

Meng Liu and co-authors
